# Retracted: Effect of Application of Treadmill Training on Metabolic Control and Vitamin D Level in Saudi Patients with Type 2 Diabetes Mellitus

**DOI:** 10.1155/2023/9828350

**Published:** 2023-02-05

**Authors:** Computational and Mathematical Methods in Medicine

*Computational and Mathematical Methods in Medicine* has retracted the article titled “Effect of Application of Treadmill Training on Metabolic Control and Vitamin D Level in Saudi Patients with Type 2 Diabetes Mellitus” [1] due to concerns that the peer review process has been compromised.

Following an investigation conducted by the Hindawi Research Integrity team [2], significant concerns were identified with the peer reviewers assigned to this article; the investigation has concluded that the peer review process was compromised. We therefore can no longer trust the peer review process and the article is being retracted with the agreement of the Chief Editor.

The authors do not agree to the retraction; author Lamiaa K. Elsayyad could not be reached by the publisher using the email address provided to the publisher with the article submission.
